# Beneficial bacteria activate nutrients and promote wheat growth under conditions of reduced fertilizer application

**DOI:** 10.1186/s12866-020-1708-z

**Published:** 2020-02-21

**Authors:** Juanjuan Wang, Ruochen Li, Hui Zhang, Gehong Wei, Zhefei Li

**Affiliations:** 1 State Key Laboratory of Crop Stress Biology in Arid Areas, Northwest A&F University, Yangling, Shaanxi China; 2 Shaanxi Key Laboratory of Agricultural and Environmental Microbiology, College of Life Science, Northwest A&F University, Yangling, Shaanxi China

**Keywords:** Plant growth-promoting rhizobacteria, Phosphate-solubilizing, Potassium-solubilizing, Nitrogen fixation, Promote

## Abstract

**Background:**

Excessive application of chemical fertilizer has exerted a great threat to soil quality and the environment. The inoculation of plants with plant-growth-promoting rhizobacteria (PGPR) has emerged as a great prospect for ecosystem recovery. The aim of this work to isolate PGPRs and highlights the effect of bacterial inoculants on available N/P/K content in soil and on the growth of wheat under conditions of reduced fertilizer application.

**Results:**

Thirty-nine PGPRs were isolated and tested for their growth-promoting potential. Thirteen isolates had nitrogen fixation ability, of which N9 (*Azotobacter chroococcum*) had the highest acetylene reduction activity of 156.26 nmol/gh. Eleven isolates had efficient phosphate solubilizing ability, of which P5 (*Klebsiella variicola*) released the most available phosphorus in liquid medium (231.68 mg/L). Fifteen isolates had efficient potassium solubilizing ability, of which K13 (*Rhizobium larrymoorei*) released the most available potassium in liquid medium (224.66 mg/L). In culture medium supplemented with tryptophan, P9 (*Klebsiella pneumoniae*) produced the greatest amount of IAA. Inoculation with the bacterial combination K14 + 176 + P9 + N8 + P5 increased the alkali-hydrolysed nitrogen, available phosphorus and available potassium in the soil by 49.46, 99.51 and 19.38%, respectively, and enhanced the N, P, and K content of wheat by 97.7, 96.4 and 42.1%, respectively. Moreover, reducing fertilizer application by 25% did not decrease the available nitrogen, phosphorus, and potassium in the soil and N/P/K content, plant height, and dry weight of wheat.

**Conclusions:**

The bacterial combination K14 + 176 + P9 + N8 + P5 is superior candidates for biofertilizers that may reduce chemical fertilizer application without influencing the normal growth of wheat.

## Background

China has the largest population in the world.The application of chemical fertilizer has played a key role in increasing grain production in China to produce sufficient food for a very large population with limited arable land [1]. In recent decades, to meet the nutritional needs of a rapidly growing population, China’s the annual total demand for chemical fertilizers has shown an upward trend; that country has become the largest manufacturer and consumer of chemical fertilizers, accounting for one-third of the global total consumption [2]. For example, from 1997 to 2006, the average application of nitrogen fertilizer for rice increased from 145 kg/ha^− 1^ to 300 kg/ha^− 1^ in the Taihu region [3], a significantly larger increase than in countries outside China. However, the utilization efficiency of these fertilizers is only 30–40%, indicating that most applied chemical fertilizer is lost by different pathways, such as ammonia (NH_3_) volatilization and leaching [4–6].

Repeated overuse of chemical fertilizer can have a negative effect on soil quality and soil microbial community structure. Excessive fertilizer aggravates the decline of soil organic matter and fertility and accelerates soil acidification, which in turn reduces crop yield [7, 8]. Therefore, there is an urgent need to find an environmentally friendly strategy to reduce the application of chemical fertilizer and increase crop yield. In many countries, it is a widely accepted practice to enhance sustainable agricultural production by inoculating crops with plant growth-promoting rhizobacteria (PGPR).

Plant-growth-promoting rhizobacteria are free-living soil microorganisms that inhabit the rhizosphere or plant roots during plant growth and development. PGPR can promote plant growth, help prevent pathogen invasion and improve plant adaptability to abiotic or biological stresses [9–11]. The beneficial effects of PGPR on plants can be explained by different mechanisms, including (1) nitrogen fixation [12]; (2) inorganic phosphate solubilization and organic phosphate mineralization [13]; (3) production of plant growth regulators or phytohormones such as indole-3-acetic acid (IAA), cytokinins, and gibberellins [14, 15]; (4) production of siderophores, 1-amino-cyclopropane-1 -carboxylate (ACC) deaminase, and hydrogen cyanate [16, 17]; (5) and biological control of phytopathogens and insects by synthesizing antibiotics or fungicidal compounds or competiting with detrimental microorganisms [18, 19].

Agricultural problems caused by the long-term use of pesticides, fertilizers and other products have become increasingly prominent. This not only pollutes agricultural products but also causes an imbalance in the proportions of various nutrients, the destruction of organic matter in the soil and a decrease in the structural integrity and properties of aggregates, leading to soil compaction, salinization and disease aggravation. However, soil microorganisms are involved in various biotic activities of the soil ecosystem to promote dynamic turnover and sustainable crop production. Thus PGPR and their interactions with plants have great application prospects in ecological agriculture and sustainable agriculture. Co-inoculation of cotton with *Azotobacter chroococcum* strains can positively influence plant growth and reduce nitrogen fertilization doses by 50% [20]. Korir et al. [21] conducted experiments using a low-phosphorous soil under greenhouse conditions to examine the effect of PGPR on the nodulation and growth of common bean. The results showed that mixed inoculaiton with strains of *Bacillus megaterium* and *Rhizobium tropici* significantly increased nodule fresh weight, plant dry weight, and root dry weight by 192.2, 124.5, and 126.7%, respectively.

In northwest China, wheat is a staple food that has special regional importance. Low annual precipitation limits crop growth, and there is much emphasis on the use of large amounts of chemical fertilizer to increase wheat yields. However, information is scarce regarding the isolation of PGPR from the wheat rhizosphere and the role of PGPR in nitrogen fixation, phosphate solubilization, plant growth promotion and reduction of the need for fertilizer in wheat. This study was planned to (1) isolate PGPR from the rhizosphere/roots of wheat and screen them in vitro for plant growth promotion potential; (2) investigate the effect of PGPR on the availability of nitrogen, phosphorus and potassium in soil and the growth of wheat; and (3) evaluate the role of PGPR inoculation in reducing fertilizer input.

## Results

### Plant-growth-promoting potential

**Fig. 1:**
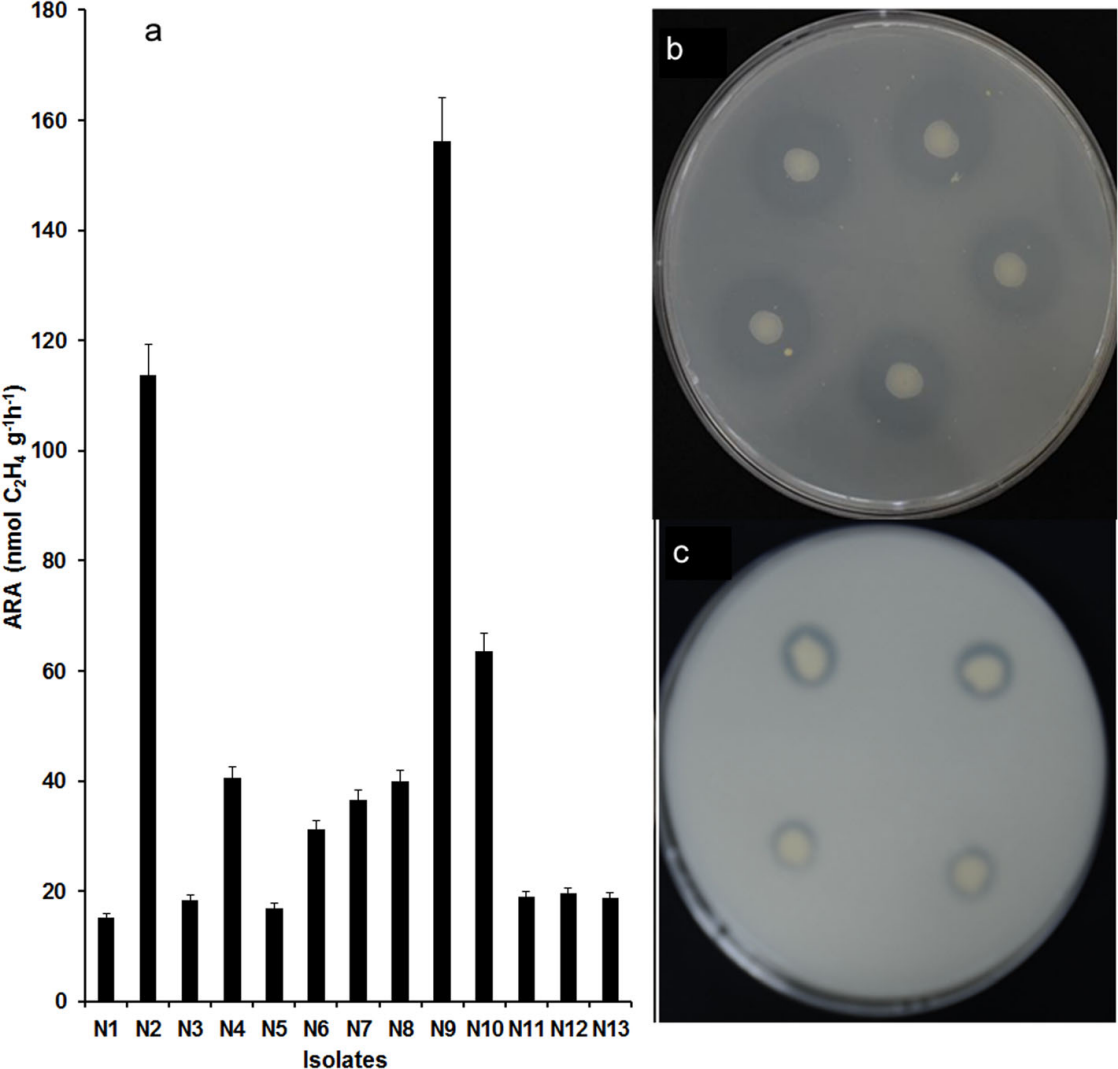
Plant growth promoting potential of bacteria. **a** Acetylene reduction activity (ARA) of thirteen isolates. N1: *Paenibacillus* SP. N2, N4, N7: *Azotobacter* sp. N3, N5: *Rheinheimera* sp. N6: *Agrobacterium* sp. N8, N11: *Pseudomonas* sp. N9: *Azotobacter chroococcum* N10: *Enterobacter* sp. N12: *Pantoea* sp. N13: *Erwinia* sp. **b** Solubilization zone of organic phosphate **c** Solubilization zone of inorganic phosphate

Among the bacterial isolates in this study, thirteen strains (N1-N13) were able to grow on nitrogen-free medium. An acetylene reduction activity (ARA) assay indicated that all thirteen isolates had nitrogenase activity, ranging from 15.14 to 156.26 nmol/gh. The nitrogenase activity of isolates N2 and N9 was significantly higher than that of all other strains, and their ARA values were 113.67 and 156.26 nmol/gh, respectively (Fig. 1a).

Eleven bacterial isolates were able to form a halo zone around the colonies by P solubilization. Four isolates (P2, P4, P6 and P7) could dissolve inorganic but not organic phosphate, three isolates (P8, P10 and P11) could dissolve organic but not inorganic phosphate and four isolates (P1, P3, P5 and P9) were able to dissolve both inorganic and organic phosphate (Table 1). P5 had the highest solubilization index (2.56) on PVK medium, while P11 had the highest solubilization index (4.57) on organic phosphorus dissolution medium (Fig. 1b, c and Table 1). Quantitative determination showed that the range of inorganic phosphate solubilization varied from 67.18 to 231.68 mg/L, and the range of organic phosphate solubilization was between 13.67 and 40.40 mg/L (Table 1).

**Table 1:** Phosphorus releasing ability of PGPR

Isolates	PGPR activities	Inorganic phosphate		Organic phosphate		IAA production	pH
		SI	P content(mg/L)	SI	P content (mg/L)		
P 1 (*Klebsilla pneumoniae*)	Organic and inorganic P solubilizing, IAA production	1.73	172.28 ± 3.15	+		++	5.32
P 2 (*Klebsiella* sp.)	Inorganic P solubilizing, IAA production	1.52	109.25 ± 3.15	–		++	5.46
P 3 (*Enterobacter asburiae*)	Organic and inorganic P solubilizing, IAA production	2.38	189.19 ± 3.91	+		+	5.13
P 4 (*Raoultella* sp*.*)	Inorganic P solubilizing, IAA production	1.17	95.79 ± 3.78	–		+	5.78
P 5 (*Klebsiella variicola*)	Organic and inorganic P solubilizing, IAA production	2.56	231.68 ± 3.13	+		+++	4.96
P 6 (*Agrobacterium* sp.)	Inorganic P solubilizing	1.32	67.18 ± 3.74	–		–	6.32
P 7 (*Rhizobium* sp.)	Inorganic P solubilizing, IAA production	1.89	140.21 ± 3.81	–		+	5.96
P 8 (*Enterobacter* sp.)	Organic P solubilizing, IAA production	–		2.87	13.64 ± 1.00	+	6.78
P 9 (*Klebsiella* sp.)	Organic and inorganic P solubilizing, IAA production	+		2.50	29.93 ± 2.47	+++	5.63
P 10 (*Enterobacter* sp.)	Organic P solubilizing	–		2.00	8.49 ± 0. 7	–	6.37
P 11 (*Comamonas* SP.)	Organic P solubilizing, IAA production	–		4.57	40.40 ± 1.90	+	5.26
Control		–	1.23 ± 0.12	–	0.67 ± 0.07	–	7.02

Note: + means the isolate can solubilize organic, inorganic phosphate or produce IAA, − means the isolate can not solubilize organic/inorganic phosphate

Fifteen bacterial isolates were able to form clear zone on Aleksandrov medium. After inoculating these 15 strains of bacteria, the soluble potassium content in the supernatant of culture medium ranged from 38.55 to 224.66 mg/L. K13 strain had the strongest potassium releasing ability (Table 2). Moreover, among 39 plant growth promotion bacteria, except isolates P6, P10 and K15, the others could produced IAA in the presence of tryptophan. But the amount of IAA produced by them is quite different. P9 produced maximum IAA followed by P5, K7, K5, P1 and K13 (Tables 1, 2, 3). Of all the isolates, only K14 had strong cellulose degradation ability (Additional file 3: Table S1).

**Table 2:** Potassium releasing ability of PGPR

Isolates	PGPR activities	SI	K content(mg/L)	IAA production	pH
K1 (*Klebsiella variicola*)	K and P solubilizing, IAA production	2.78	95.97 ± 3.75	++	4.49
K2 (*Enterobacter* sp.)	K and P solubilizing, IAA production	2.86	94.64 ± 3.00	+	4.66
K3 (*Raoultella* sp*.*)	K and P solubilizing, IAA production	2.79	83.80 ± 2.40	+	4.97
K4 (*Klebsiella variicola*)	K and P solubilizing, IAA production	2.65	57.20 ± 1.62	++	6.11
K5 (*Klebsiella variicola*)	K and P solubilizing, IAA production	1.39	64.16 ± 2.19	++	5.15
K6 (*Klebsilla pneumoniae*)	K and P solubilizing, IAA production	1.57	38.55 ± 2.82	++	6.32
K7 (*Klebsiella* sp.)	K and P solubilizing, IAA production	1.26	39.71 ± 1.23	+++	6.18
K8 (*Raoultella* sp*.*)	K and P solubilizing, IAA production	2.02	67.36 ± 2.55	+	5.29
K9 (*Raoultella* sp*.*)	K and P solubilizing, IAA production	1.88	49.84 ± 2.77	+	5.03
K10 (*Pseudomonas* sp.)	K and P solubilizing, IAA production	3.59	193.33 ± 3.87	+	4.42
K11 (*Advenella* sp.)	K and P solubilizing, IAA production	2.97	117.20 ± 3.50	+	4.78
K12 (*Agrobacterium* sp.)	K and P solubilizing, IAA production	3.02	154.78 ± 3.11	+	4.31
K13 (*Rhizobium* sp.)	K and P solubilizing, IAA production	3.85	224.66 ± 2.69	++	4.03
K14 (*Bacillus* sp.)	K and P solubilizing, IAA production	1.93	81.13± 3.50	+	5.22
K15 (*Sphinqomonas* sp.)	K and P solubilizing	9.92	104.59 ± 2.74	–	5.45
Control	–	–	31.96 ± 1.60	–	7.20

**Table 3:** PGPR activities of nitrogen fixing bacteria

Isolates	PGPR activities	IAA	Isolates	PGPR activities	IAA
N1 (*Paenibacillus* SP.)	Nitrogen fixation, IAA production	+	N8 (*Pseudomonas* sp.)	Nitrogen fixation, IAA production	+
N2 (*Azotobacter* sp.)	Nitrogen fixation, IAA production	+	N9 (*Azotobacter chroococcum*)	Nitrogen fixation, IAA production	+
N3 (*Rheinheimera* sp.)	Nitrogen fixation, IAA production	+	N10 (*Enterobacter* sp.)	Nitrogen fixation, IAA production	++
N4 (*Azotobacter* sp.)	Nitrogen fixation, IAA production	+	N11 (*Pseudomonas* sp.)	Nitrogen fixation, IAA production	+
N5 (*Rheinheimera* sp.)	Nitrogen fixation, IAA production	+	N12 (*Pantoea* sp.)	Nitrogen fixation, IAA production	++
N6 (*Agrobacterium* sp.)	Nitrogen fixation, IAA production	+	N13 (*Erwinia* sp.)	Nitrogen fixation, IAA production	+
N7 (*Azotobacter* sp.)	Nitrogen fixation, IAA production	+			

### Identification of bacteria

Thirty-nine rhizosphere/endophytic bacteria with potential growth-promoting ability were isolated in vitro. Based on the sequencing results of the 16S rRNA gene, these bacterial isolates belonged to 15 genera (Additional file 3: Table S1). The isolate N9 (GenBank accession number: MN658515) had the closest genetic relationship with *Azotobacter chroococcum* IAM12666, and its 16S rDNA sequence homology was 99.93%. The isolate N8 (Accession number: MN700634) showed 97.88% similarity with *Pseudomonas furukawaii* KF707. The strain P9 (Accession number: NR_036794) had 100% similarity with *Klebsiella pneumoniae* ATCC13884. The isolate K14 (Accession number: MN704638) had 99.34% similarity with *Bacillus niacini* IFO 15566. The isolates P5 (Accession number: MN658477), K10 (Accession number: MN704376), and K13 (Accession number: MN662624) had the closest genetic relationship with *Klebsiella variicola*, *Pseudomonas migulae*, and *Rhizobium larrymoorei* in the phylogenetic tree based on 16S rRNA gene sequences reconstructed using MEGA (version 6), respectively (Additional file 1: Figure S1).

### Plant growth parameters

**Fig. 2:**
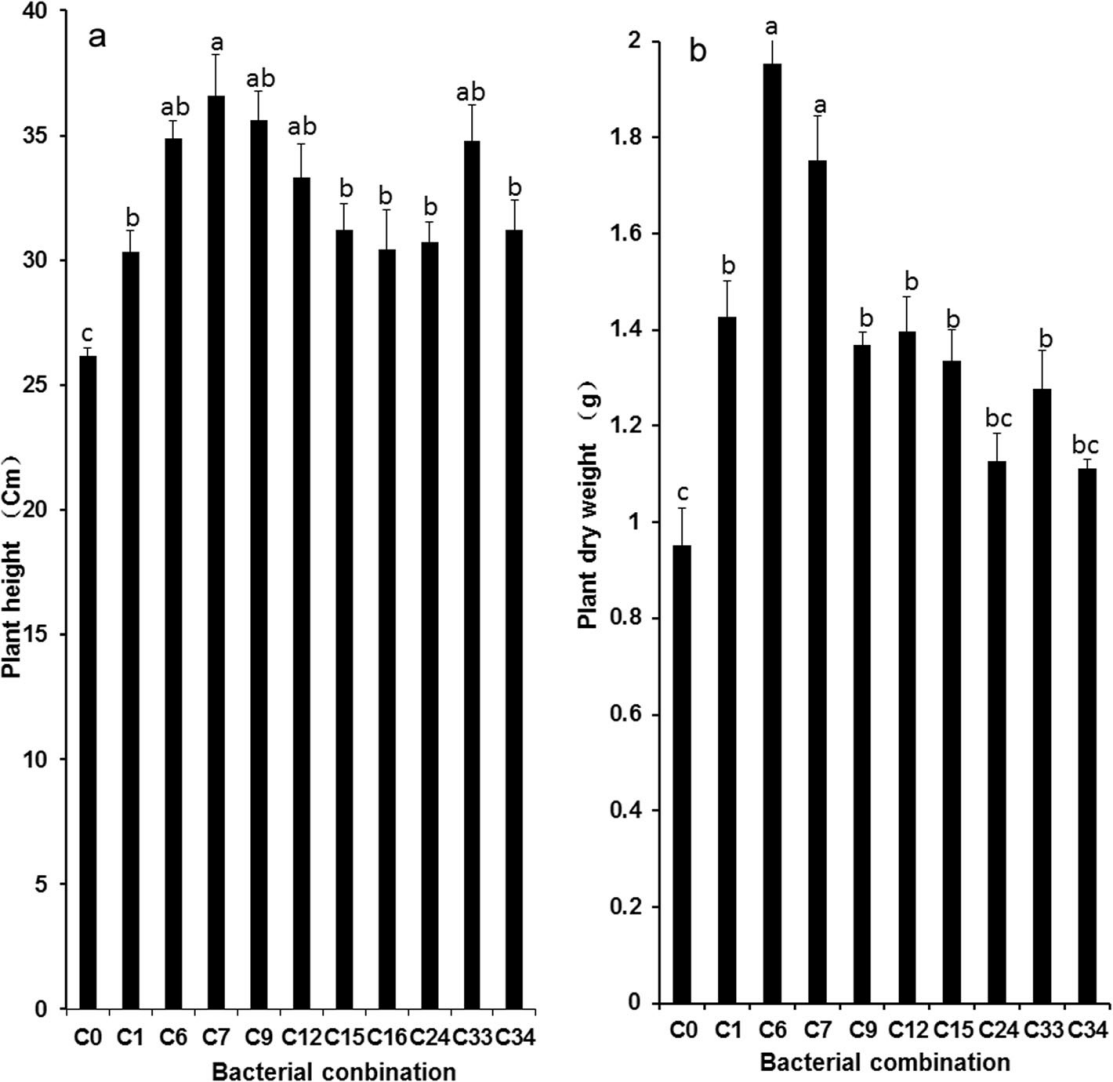
The effect of bacterial combination on **a** plant height and **b** plant dry biomass of wheat. Values are means ± SE. abcd letters on the bars denote differences on the basis of a t-test (*p* < 0.05). C0: Control C1: K14 + 176 + P9 + N8 + K10 C6: K14 + 176 + P9 + N8 + P5 C7: K14 + 176 + P9 + N9 + K10 C9: K14 + 176 + P9 + N9 + K13 C12: K14 + 176 + P9 + N9 + P5 C15: K14 + 176 + P9 + N10 + K13 C16: K14 + 176 + P9 + N10 + P1 C24: K14 + 176 + P11 + N8 + P5 C33: K14 + 176 + P11 + N10 + K13 C34: K14 + 176 + P11 + N10 + P1

To evaluate the growth-promoting effect of PGPR on wheat, 12 isolates with strong growth-promoting ability and one growth-promoting bacterium “176” (*Agrobacterium tumefaciens* CCNWGS0286) preserved in our laboratory were inoculated into plant roots. However, there was no difference in wheat plant height among 13 single-inoculated treatments and the non-inoculated treatment. Therefore, the isolates with nitrogen fixation, inorganic/organic phosphate and potassium-releasing ability and strain 176 were combined. Ten out of thirty-six PGPRs combinations significantly increased the plant height of wheat. Meanwhile, eight combinations enhanced the dry weight of wheat (Fig. 2). K14 (*Bacillus* sp.) + 176 (*Agrobacterium tumefaciens*) + P9 (*Klebsiella pneumoniae*) + N8 (*Pseudomonas* sp.) + P5 (*Klebsiella variicola*) and K14 (*Bacillus* sp.) + 176 (*Agrobacterium tumefaciens*) + P9 (*Klebsiella pneumoniae*) + N9 (*Azotobacter chroococcum*) + K10 (*Pseudomonas* sp.) maximized the dry weight and height of wheat. Shoot height and plant dry mass were increased by 18.59 and 105% (*P* < 0.05), respectively, in plants inoculated with K14 + 176 + P9 + N8 + P5 and K14 + 176 + P9 + N9 + K10 group compared to plants without inoculum (Fig. 2 and Additional file 3: Table S2).

### Determination of nutrients in rhizosphere soil

Ten bacterial combinations with good growth-promoting effects were inoculated into wheat roots, and the available nitrogen, phosphorus and potassium contents in the soil were determined after 80 days of plant growth. Compared with the control, the alkali-hydrolysed nitrogen, available phosphorus and available potassium in all inoculated soil were increased to varying degrees. Among the combinations K14 (*Bacillus* sp.) + 176 (*Agrobacterium tumefaciens*) + P9 (*Klebsiella pneumoniae*) + N8 (*Pseudomonas* sp.) + P5 (*Klebsiella variicola*) had the most notable effect on soil nutrient improvement. The alkali-hydrolysed nitrogen, available phosphorus and available potassium increased by 49.46, 99.51 and 19.38%, respectively (Table 4).

**Table 4:** The effect of bacterial combination on available N/P/K content in soil

	Combination	Alkali-hydrolyzed nitrogen (mg/kg soil)	Available P (mg/kg soil)	Available K (mg/kg soil)
C0	Control	12.13 ± 0.2 ^c^	7.48 ± 0.8^c^	87.47 ± 2.1^d^
C1	K14 + 176 + P9 + N8 + K10	18.71 ± 1.1 ^b^	11.69 ± 1.4^b^	95.79 ± 2.9^bcd^
C6	K14 + 176 + P9 + N8 + P5	21.98 ± 1.4 ^a^	14.92 ± 1.1^a^	104.42 ± 2.8^a^
C7	K14 + 176 + P9 + N9 + K10	19.28 ± 1.5 ^b^	13.09 ± 0.4^ab^	98.24 ± 2.8^abc^
C9	K14 + 176 + P9 + N9 + K13	19.55 ± 0.9 ^b^	14.19 ± 1.1^ab^	100.51 ± 1.0^ab^
C12	K14 + 176 + P9 + N9 + P5	18.71 ± 0.8 ^b^	11.49 ± 1.0^b^	90.38 ± 6.3^cd^
C15	K14 + 176 + P9 + N10 + K13	18.01 ± 1.4 ^b^	12.00 ± 1.2^ab^	93.86 ± 4.4^bcd^
C16	K14 + 176 + P9 + N10 + P1	18.13 ± 0.9 ^b^	12.63 ± 1.1^ab^	89.20 ± 5.2^cd^
C24	K14 + 176 + P11 + N8 + P5	14.51 ± 1.6 ^c^	11.47 ± 1.2 ^b^	91.33 ± 1.5^bcd^
C33	K14 + 176 + P11 + N10 + K13	14.76 ± 0.6 ^c^	11.18 ± 0.9 ^b^	86.51 ± 2.5^d^
C34	K14 + 176 + P11 + N10 + P1	14.51 ± 1.6 ^c^	11.72 ± 0.9 ^b^	93.27 ± 4.5^bcd^

Values are means ± SE. abcd letters on the bars denote differences on the basis of a t-test (*p* < 0.05)

### Determination of N/P/K content in wheat

**Fig. 3:**
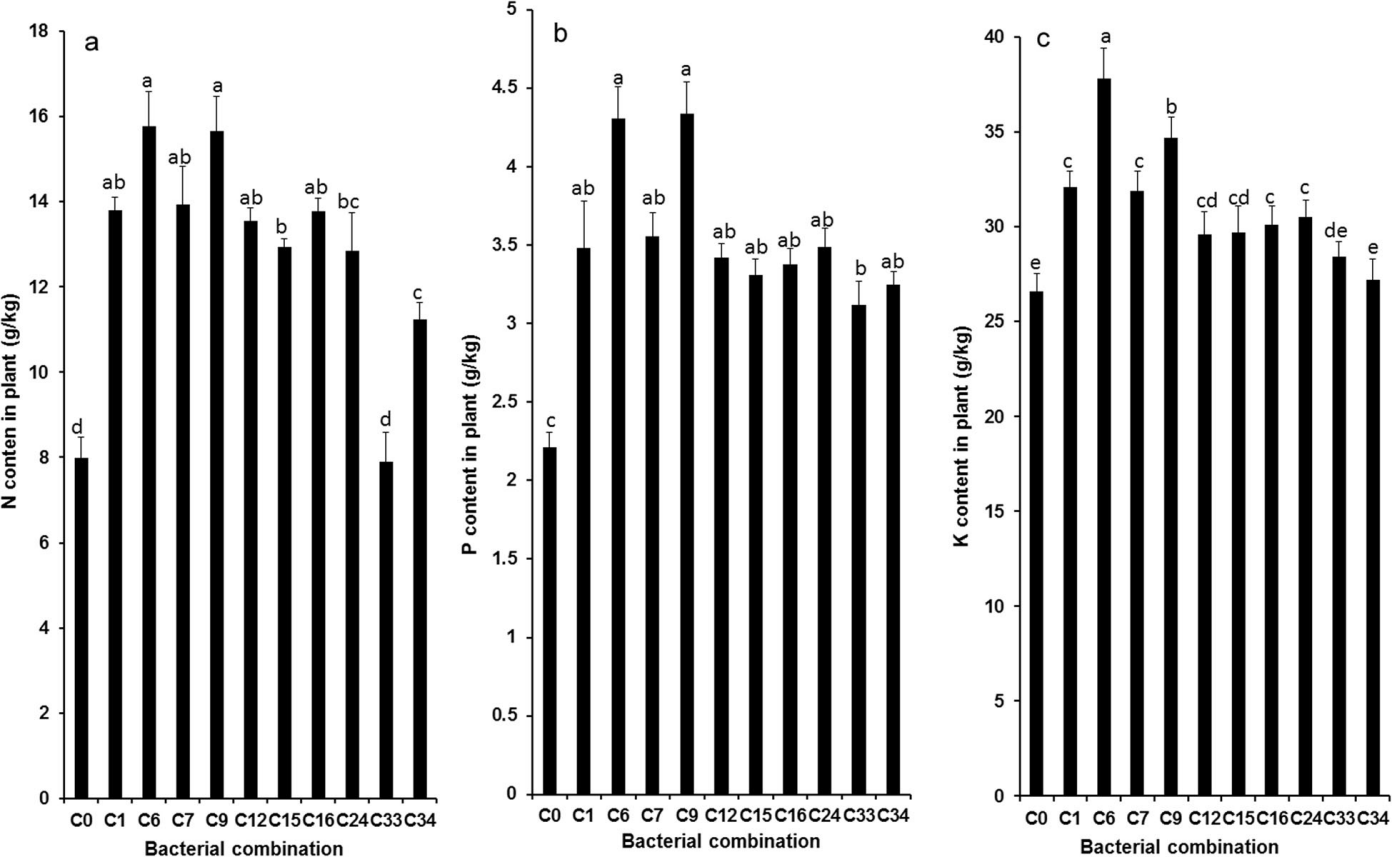
Promotion of PGPRs combination on **a** nirogen, **b** phosphorus, and **c** potassium uptake for wheat. Values are means ± SE. abcde letters on the bars denote differences on the basis of a t-test (*p* < 0.05)

In order to verify whether inoculation treatment can promote nutrient uptake by plants, the contents of nitrogen, phosphorus and potassium in wheat were determined. As shown in Fig. 3, significant increases in plant N/P/K uptake were observed when the soil was inoculated with different combinations of bacteria compared with the non-inoculated control. For wheat inoculated with different bacterial combinations, the plant N content increased from 40.7 to 97.7% (*P* < 0.05). Additionally, the P content of wheat increased from 41.2 to 96.4%, and the K content increased from 2.3 to 42.1%.

### Effect of reducing fertilizer application

**Fig. 4:**
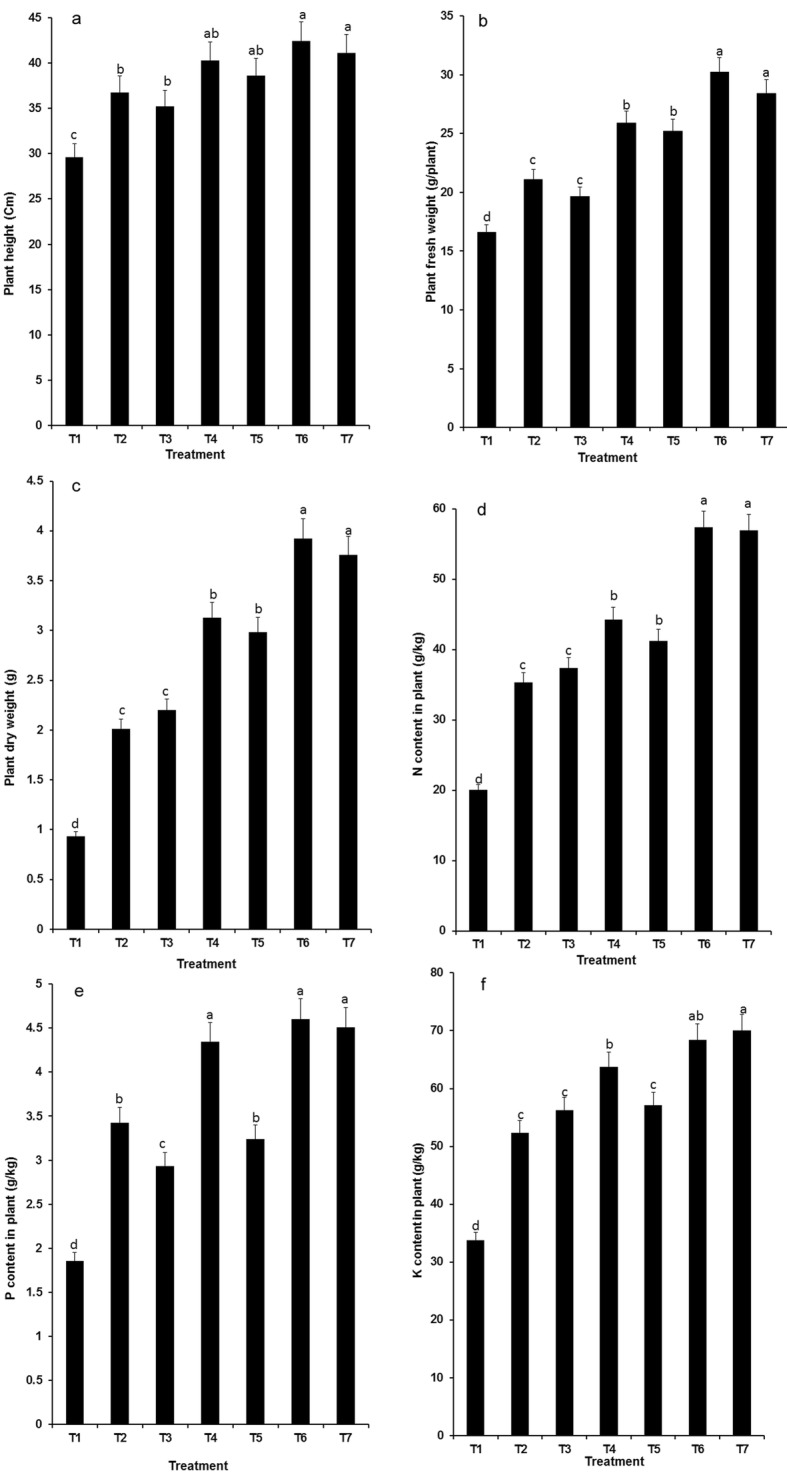
The effect of bacterial combination K14 + 176 + P9 + N8 + P5 on **a** plant height, **b** plant fresh weight, **c** plant dry weight, **d** N content in plant, **e** P content in plant, and **f** K content in plant of wheat. T1: without fertilizer, T2: PGPR combination 6, T4: 50% fertilizer + PGPR combination 6, T6: 75% fertilizer + PGPR combination 6, T7: 100% fertilizer. Values are means ± SE. abcd letters on the bars denote differences on the basis of a t-test (*p* < 0.05)

**Fig. 5:**
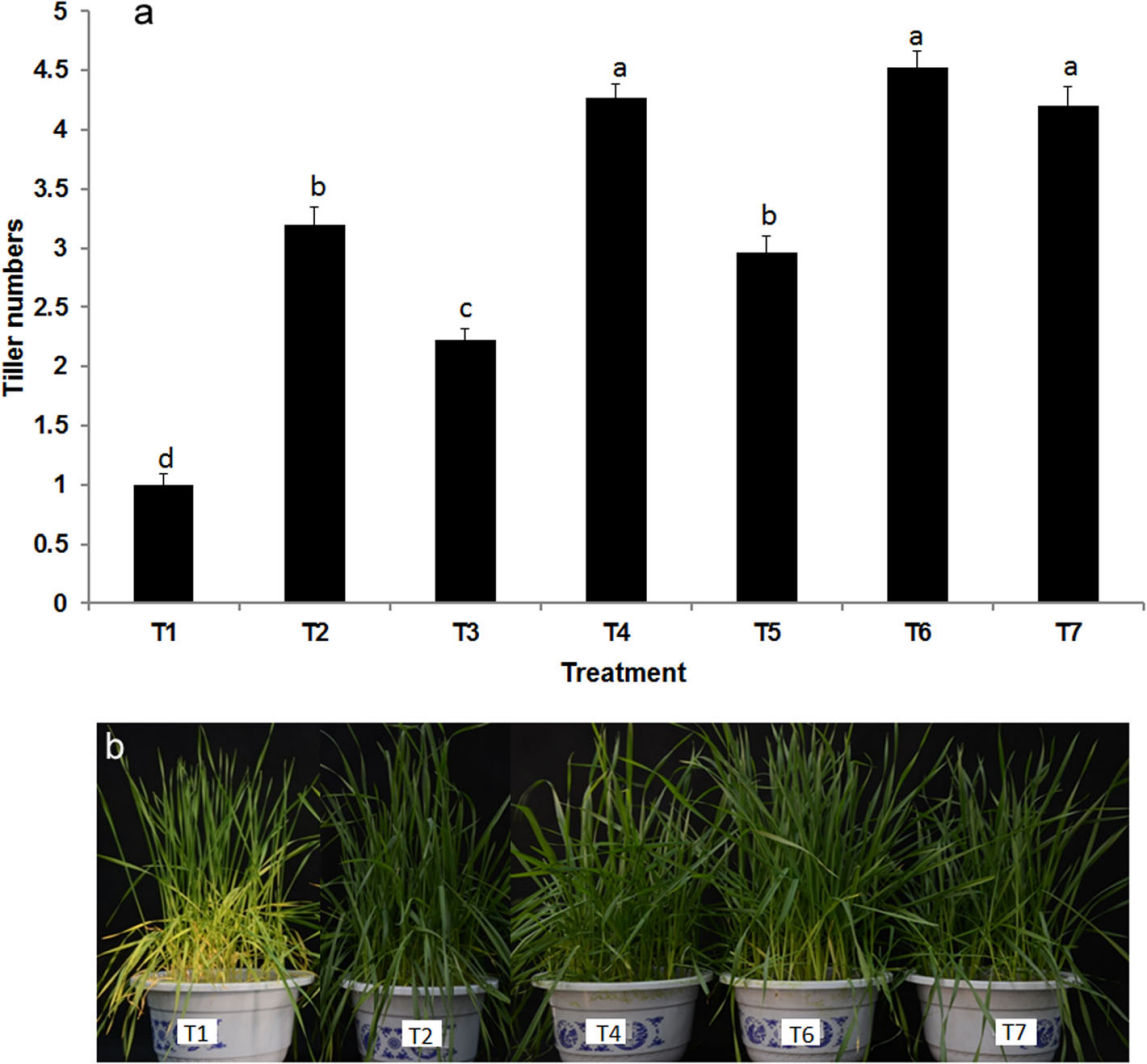
**a** The effect of bacterial combination K14 + 176 + P9 + N8 + P5 and fertilizer application on tiller number of potted wheat. Values are means ± SE. abcd letters on the bars denote differences on the basis of a t-test (*p* < 0.05). **b** Phenotype of wheat under different fertilization treatments. T1: without fertilizer, T2: PGPR combination 6, T3: 50% fertilizer, T4: 50% fertilizer + PGPR combination 6, T5: 75% fertilizer, T6: 75% fertilizer + PGPR combination 6, T7: 100% fertilizer

Since the combination of K14 + 176 + P9 + N8 + P5 could activate nutrients in soil and promote the utilization of nitrogen, phosphorus and potassium by plants, we validated whether this PGPR combination inoculation could reduce fertilizer doses. The results are shown in Figs. 4 and 5, Compared with a 100% fertilizer dose, the reduction of fertilizer application by 25, 50 and 100% significantly inhibited plant growth. When no fertilizer was added to the soil, the combined PGPR inoculation significantly increased plant height, tiller counts, fresh weight and dry weight but still did not perform as well as 100% fertilizer application without PGPR. However, there was no difference in plant height, fresh weight, tiller counts, or N/P/K content between PGPR combination + 75% fertilizer and 100% fertilizer without PGPR (Figs. 4 and 5). Compared with 100% fertilizer, PGPR combination + 75% fertilizer did not reduce available nitrogen, phosphorus or potassium in the soil (Additional file 2: Figure S2).

In sum, these results showed that the combination of K14 + 176 + P9 + N8 + P5 could promote nitrogen, phosphorus and potassium accumulation in wheat, and reduce the application of chemical fertilizer by 25%.

## Discussion

There is a very complex system of interactions among microorganisms and between microorganisms and plants in soil. Under certain conditions, plants recruit beneficial microorganisms to colonize the rhizosphere or root tissue by secreting metabolites to the soil [22]. These microorganisms can improve plant adaption to biotic and abiotic stresses [23], inhibit the growth of plant pathogens by producing antimicrobial compounds or competing with pathogens for nutrients [24], and activate nutrients in soil [25]. The nitrogen-fixing, and P/K-releasing potential of bacteria isolated from the rhizosphere and root of wheat was analysed. Finally, thirty-nine bacterial strains with plant-growth-promoting ability were isolated using different screening media. The 16S rRNA sequence analysis revealed that these bacteria belong to 15 genera (Additional file 3: Table S1). The nitrogenase activity of *Azotobacter* was much higher than that of other bacteria (Fig. 1), and strain N9, with the highest nitrogenase activity, showed 99.93% similarity with *A. chroococcum* IAM12666. The results presented in this paper are in agreement with previous works. *A. chroococcum* has been isolated from the rhizosphere or roots of many plants such as litchi, rice, and faba bean [26, 27]. The results of a study by Rodelas et al. [27] showed that the acetylene reduction activity of *Azotobacter* sp. was between 9.7 and 257.7 nmol C_2_H_4_ h^− 1^ vial^− 1^, while *A. chroococcum* isolates had a high capacity for acetylene reduction.

In addition to nitrogen, phosphorus and potassium are the other two essential plant growth-limiting nutrients. Although potassium and phosphorus are abundant elements in soil, they are mostly bound to other minerals. Approximately 95–99% of phosphorous and potassium are present in insoluble form and therefore unavailable to plants. Some soil microorganisms can dissolve otherwise insoluble phosphate and potassium. Several studies have shown that different bacterial genera, such as *Pseudomonas*, *Bacillus*, *Rhizobium*, *Agrobacterium*, *Klebsilla*, and *Erwinia*, have the potential to solubilize and release phosphorus/potassium from soil [28]. In the present work, 11 phosphate-solubilizing bacteria and 15 potassium-solubilizing bacteria were isolated from the rhizosphere and roots of wheat. These microbial isolates showed potassium solubilization index from 1.26 to 3.85 or phosphate solubilization indices from 1.17 to 4.57 on PVK or Aleksandrov medium (Tables 1 and 2). These bacterial isolates comprise 9 *Klebsilla*, 4 *Enterobacter*, 4 *Raoultella*, 2 *Rhizobium*, 2 *Agrobacterium*, 1 *Bacillus*, 1 *Advenella*, 1 *Microbacterium*, 1 *Comamonas*, and 1 *Pseudomonas* isolate (Additional file 3: Table S1), this is consistent with previous studies. Maliha et al. [29] showed that bacteria released organic acids and decreased the pH of the rhizosphere, which led to the dissolution of the mineral phosphate. The selected bacterial strains were grown using a broth culture method, and the phosphorus/potassium content and pH of the culture medium were analysed quantitatively. Compared with the non-inoculated control, the inoculated treatment showed significantly increased phosphorus/potassium content in broth and reduced median pH. Meanwhile, the more pH decreased, the larger the solubilization index and the more phosphorus/potassium content in broth was observed. Potassium-solubilizing bacteria can also dissolve tricalcium phosphate in medium (Tables 1 and 2 and Additional file 3: Table S1). The results indicated that the bacteria we isolated dissolve insoluble phosphate/potassium mainly by producing organic acids.

In addition to focusing on biological nitrogen fixation and phosphate/potassium solubilization, we investigated the IAA-producing potential of in these bacterial isolates. The results showed that most of the isolates could synthesize IAA, but their production rates were quite different. This variation is due to the different synthetic pathways, key genes and regulatory strategies of different bacteria [30]. Bacteria isolated from the rhizosphere of soybean and rice have been shown to produce 10.54 to 84.72 mg/kg IAA [31, 32]. The strain P9 (P-solubilizing bacteria) showed the highest IAA production, followed by the strains P5 and K7 (Tables 1, 2, and 3). The measured production of IAA was higher in this work than in previous reports. This suggested that these isolates could serve as efficient biofertilizer candidates for activating phosphorus/potassium and simultaneously promoting crop growth.

It is important to consider that the growth-promoting performance of bacteria will be influenced by biotic and abiotic factors in soil. We combined bacteria with different functions and performed experiments in a greenhouse. The results of pot-based experiments showed that ten combinations significantly increased the available phosphorus/potassium and alkali hydrolysed nitrogen in the soil, and the plant height, dry biomass, and N/P/K content of wheat also increased after inoculation with these ten bacterial combinations. Among them, the combination K14 + 176 + P9 + N8 + P5 had the best growth-promoting effect (Fig. 4). In combination K14 + 176 + P9 + N8 + P5 with better growth promoting effect, the isolates P5, N8, P9 and K14 had the closest genetic relationship with *Klebsiella Klebsiella variicola*, *Pseudomonas furukawaii*, *Klebsiella pneumoniae* and *Bacillus niacini*, respectively. Through analysis the genome of the strain 176, two possible pathways for IAA synthesizing have been found. The genes ATCR1_06021 and ATCR1_10873, ATCR1_02965, ATCR1_10883, ATCR1_10878, ATCR1_02970, and ATCR1_23021 may be inovolve in the synthesis of tryptophan. Genes ATCR1_17572 (encoding amine oxidase) and ATCR1_06371 (indole-3-acetaldehyde dehydrogenase) involved in TAM (tryptamine) pathway, while ATCR1_17003 (nitrilase) and ATCR1_07569 (monooxygenase) involved in IAM (indole-3-acetamide) pathway. Previous study has confirmed that excessive production of IAA by strain 176 is an important reason for plant growth-promoting [33]. In *K. variicola* DX120E, the genome contains *nif* gene cluster, indole-3-pyruvate decarboxylase, and siderophore enterobactin synthesis genes (*ent*ABCDEF) which contribute to N_2_ fixation, indole-3-acetic acid production, siderophore production, and phosphate solubilization. Besides ferric iron uptake transcriptional regulator gene *fur* and indole-3-glycerol phosphate synthase gene *trp*C, there are 10 inorganic phosphorus dissolving genes (from *pqq*A to *pqq*F) in *P. furukawaii* KF707^T^ genome. This strain also encodes antioxidant enzymes, such as superoxide dismutase and catalase, which have the ability to remove free radicals under stresses. The genome of *B*. *niacini* NBRC 15566^T^ contains 13 genes involved in iron transport and ferric uptake regulation, and there are *trp*C gene (encoding indole-3-glycerol phosphate synthase) and 4 phospholipase genes. Thus these growth-promoting genes may be closely related to the increase of available N, P, K in soil and plant growth (Fig. 1, Tables 1, 2, and 3). Thus we speculated that the improvement of plant growth can be attributed to nitrogen fixation and phosphate/potassium solubilization by PGPR, which provides great deal of nutrition for plant development [34]. Furthermore, IAA can be produced by inoculated PGPR. IAA is involved in cell division, root growth, and stem elongation, increasing the surface area of roots such that plants con obtain additional water and nutrients [35]. A large number of studies have focused on the role of PGPR in promoting crop growth [36, 37]. However, bacteria are not a complete substitute for fertilizers. In the present study, the potential of PGPR in reducing fertilizer application was also investigated. The plant dry weight, N/P/K content and tiller counts were not affected by a 25% decrease in fertilizer after inoculation with the combination K14 + 176 + P9 + N8 + P5. In conclusion, these PGPR can be used as biofertilizer and have broad application prospects in sustainable agricultural development.

## Conclusions

These results clearly showed that N_2_-fixing, P-solubilizing, K-solubilizing and IAA-producing bacteria can significantly increase available nitrogen, phosphorus and potassium in soil. A combination of growth-promoting bacteria with different functions effectively improved N/P/K uptake and plant growth in wheat. These bacterial isolates allow the use of lower chemical fertilizer doses than traditional fertilization strategies and have greater application potential in the field. However, future studies are needed to investigate other conditions, locations and crops to verify the reliability of the present study.

## Methods

### Isolation of bacteria

Samples of wheat were obtained from fields in which wheat had been cultivated continuously for more than 10 years in Yangling, China. Plants with rhizosphere soil were placed in sterile plastic bags and brought back to the laboratory. For isolation of rhizosphere bacteria, 1 g of rhizospheric soil was taken in 10 ml of sterile water and shaked for 10 min. 1 *g* of fresh wheat roots were washed under running tap water and surface sterilized with 4% NaOCl for 1 min. After washing six times with sterilized distilled water, the roots were ground with a sterilized mortar and pestle. These two suspension were then serially diluted to 10^− 8^ grades, 0.1 ml of each dilution of 1 × 10^− 6^, 1 × 10^− 7^, and 1 × 10^− 8^ dilution was spreaded on TY agar plates (Tryptone 5 g, Yeast extract 3 g, CaCl_2_ 0.2 g, Agar 15 g, distilled water 1000 ml). The plates were incubated at 28C for 5 days. Isolates showing different colony characters were selected, purified and stored in liquid TY medium containing 20% glycerol at − 80 °C.

### Screening for plant-growth-promoting traits

#### Nitrogen fixation

The purified colonies were streaked on Ashby agar (sucrose 10 g, KH_2_PO_4_ 0.2 g, NaCl 0.2 g, MgSO_4_ 0.2 g, K_2_SO_4_ 0.2 g, CaCO_3_ 5 g, agar 15 g, distilled water 1000 ml) and incubated at 30 °C for 7 days. The sticky colonies were inoculated into Ashby solid medium vials and incubated at 28 °C for 24 h. Five hundred microliters of air was replaced with 500 μL of acetylene, and the vials were incubated at 28 °C for 24 h. Then 600 μL of gas was extracted from vials and the ethylene content was analysed with a gas chromatograph (Shimadzu GC-17A, Japan) equipped with an H-flame ionization detector using methods described by Hardy et al. [38]. At the same time, the bacteria on surface of the solid medium in the vials were washed down, dried and weighed. The acetylene reducing activity (ARA) was calculated as follows:

$$ ARA=\frac{Ethylene\ production\ (nmol)}{Bacteria\ weight\ (g)\times Reaction\ tiem\ (h)} $$

#### Solubilization of phosphate and potassium

Each bacterial colony was inoculated on PVK medium [39], organic phosphorus dissolution medium [glucose 10 g, (NH_4_)_2_SO_4_ 0.5 g, NaCl 0.3 g, KCl 0.3 g, FeSO_4_ 0.03 g, MnSO_4_ 0.03 g, CaCO_3_ 5 g, lecithin 0.2 g (Aladdin Biochemical Technology Co., Ltd., China), agar 15 g and distilled water 1000 ml], and Aleksandrov medium [glucose 5 g, MgSO_4_ 0.5 g, CaCO_3_ 0.1 g, FeCl_3_ 0.006 g, Ca_3_PO_4_ 2.0 g (Kemiou Chemical Reagent Co., Ltd., China), Potassium aluminium silicate 3.0 g (Daiquan Fine Chemical & Technology Co., Ltd., China), agar 15 g and distilled water 1000 ml], respectively. The plates were incubated at 28 °C for 7 days and observed for the clear zone around the colonies. The solubilization index (SI) was calculated as follows:

$$ SI= Diameter\ of\ zone\ of\ clearance/ Diameter\ of\ colony $$

Then, the phosphate/potassium-soluble strains were inoculated into the corresponding liquid medium and incubated at 28 °C, 180 rpm for 5 days. The pH value of solution was measured by PHS-3C pH meter (INESA Scientific Instrument Co., Ltd, China).The P concentration in the solution was determined using the molybdovanadate method [40]. The K concentration in the solution was estimated using atomic absorption spectrometry [41].

#### Production of IAA

Bacterial isolates were grown in TY supplemented with tryptophan (500 mg/L) and incubated at 28 °C for 48 h on a rotary shaker. Cultures were centrifuged at 12,000 *g* for 10 min. A 1 ml volume of supernatant was placed into an EP tube, and an equal volume of Salkowski reagent was added and the optical density of 530 nm (OD530) of solution was measured after 30 min of reaction in dark. The different concentration of IAA (0, 20, 40, 60, 80, 100, 120, 140, 160 and 180 mg/L) was prepared. OD530 of each concentration IAA solution was determined and the standard curve was drawn. Then the IAA content of bacterial culture medium was calculated based on standard curve.

#### Determination of cellulose degradation

Each bacterial colone was inoculated on cellulolytic culture medium (sodium carboxymethyl cellulose 5 g, yeast extract 2 g, KH_2_PO_4_, 0.5 g MgSO_4_ 0.5 g, agar 15 g and distilled water 1000 ml) and incubated at 28 °C for 3 days. Then, colonies were stained with 1 mg/mL Congo red dye for 15 min and decolorized with 1 mol/L NaCl for 30 min. Colonies with clear zones indicated that the bacteria had the ability to degrade cellulose.

#### Molecular characterization of the PGPR strains

The total genomic DNA of 39 bacterial strains was extracted according to the method of Wilson & Carson [42]. The 16S rRNA region was amplified using forward primer 27F (5-AGAGTTTGATCC TGGCTCAG-3) and reverse primer 1492R (5-TACGGCTACCTTGTTACGACTT-3). The resulting products were confirmed on a 1% agarose gel, and then the correct PCR products were sequenced Sangon Biotech (Shanghai) Co., Ltd., China. The obtained gene sequences were analysed using the BLASTN program.

#### Plant growth promotion

The top layer of the soil, 0–20 cm deep, was collected from abandoned farmland, air dried, passed through a 2 mm sieve and thoroughly homogenized. Pots measuring 23 cm in diameter and 17 cm in height were filled with 3 kg of soil each. Seeds of the wheat (*Triticum aestivum* L.) variety Xiaoyan 22 were purchased from Yangling Jinnuo seed Co., Ltd. in China. The seeds were surface sterilized with a 3% sodium hypochlorite solution for 10 mins, followed by six rinses with deionized distilled water. Sterilized wheat seeds were planted in the pots. After the wheat seedlings had been growing for 1 week, 10 plants were retained in each pot, and 10 mL of a combination of 5 PGPRs (contains 2 mL each PGPR with 10^8^ colony formation unit) was inoculated into plant roots. K14 has a strong ability to degrade cellulose, 176 is a known growth-promoting bacterium preserved in our laboratory; all the PGPR combinations contain these two bacterial strains. The other three strains were selected from phosphorus-solubilizing (P1, P5, P9 and P11), nitrogen-fixing (N8, N9 and N10) and potassium-solubilizing bacteria (K10 and K13). This results in 36 inoculation combinations. Uninoculated pots were included as negative controls. All pots were placed in a greenhouse with a 16/8 h photoperiod (light/dark) and at 25 ± 1 °C for 80 days. The plants were watered with 250 mL water every 3 days during the experiment. The plants were harvested after 80 days, and the fresh weight, dry weight, shoot length, and N/P/K content of the plants and the available N/P/K concentrations in the soil were determined. Alkali-hydrolyzable N was analyzed by Kjeldahl digestion methods on a Kjeltec 8400 analyzer unit (Foss-Tecator AB, Hoganas, Sweden), available P in plant samples and soils were estimated by molybdovanadate method; whereas available K was analyzed using an atomic absorption spectrophotometer (Hitachi, Tokyo, Japan).

#### Experiments on reducing fertilizer application

The soil was collected from farmland. Soil treatment, PGPR inoculation and planting were the same as in the “Plant growth promotion” section. The experimental treatment included pots with no fertilizer and PGPRs mixture (T1); pots inoculated with PGPRs mixture (T2); pots supplied with 50% (T3), 75% (T5) or 100% fertilizer (T7); pots inoculated with PGPRs mixture and supplied with 50% (T4) or 75% fertilizer (T6). The experiment was set up with 8 replicate pots per treatment. The doses of chemical fertilizer were based on a dose commonly used by farmers (105 kg N, 112.5 kg P_2_O_5_, and 120 kg K_2_O per hectare). Plants were harvested after 3 months, and the fresh weight, dry weight, shoot length, and tiller count of the plants were determined, along with the available N/P/K concentrations of the soil.

### Data analyses

All experiments in the present study were performed in at least three replicates. The data were analysed using SPSS software version 23.0 (Armonk, NY, USA). The significant differences among various treatments were compared using Fisher’s protected LSD test with *P* ≤ 0.05.

## Supplementary information

**Additional file 1: Figure S1.** Neighbour-joining tree based on 16S rRNA gene sequences of PGPRs.

**Additional file 2: Figure S2.** The effect of bacterial combination K14 + 176 + P9 + N8 + P5 and fertilizer application on N/P/K content in soil. Values are means ± SE. abcd letters on the bars denote differences on the basis of a t-test (*p* < 0.05).

**Additional file 3:** Identification of bacteria and its promoting effect on plants.

## Data Availability

The datasets supporting the conclusions of this article are included within the article. All the bacterial strains can be obtained from the lab of Gehong Wei upon request.

## Abbreviations

ARA: Acetylene reduction activity
IAA: Indole-3-acetic acid
PGPR: Plant growth promoting rhizobacteria
SI: Solubilization index
