# Supplementary material for: Beneficial bacteria activate nutrients and promote wheat growth under conditions of reduced fertilizer application
Source: BMC Microbiol. 2020 Feb 21;20:38. doi: 10.1186/s12866-020-1708-z (PMC7035779; doi:10.1186/s12866-020-1708-z)
Supplement: Supplementary file 3 — Additional file 3: Identification of bacteria and its promoting effect on plants. [file 12866_2020_1708_MOESM3_ESM.docx]

Table S1 Molecular identification PGPR based on 16S rDNA sequencing

| Isolates | Genus | | Nitrogen fixation | Inorganic phosphate solubilization | | Organic Phosphate solubilization | Potassium solubilization | cellulose degradation |
| --- | --- | --- | --- | --- | --- | --- | --- | --- |
| N1 | *Paenibacillus mucilaqinosus* | | + | N | N | | N | - |
| N2 | *Azotobacter* sp. | + | | N | N | | N | - |
| N3 | *Rheinheimera* sp. | + | | N | N | | N | - |
| N4 | *Azotobacter* sp. | + | | N | N | | N | - |
| N5 | *Rheinheimera* sp. | + | | N | N | | N | - |
| N6 | *Agrobacterium* sp. | + | | N | N | | N | - |
| N7 | *Azotobacter* sp. | + | | N | N | | N | - |
| N8 | *Pseudomonas* sp. | + | | N | N | | N | - |
| N9 | *Azotobacter chroococcum* | + | | N | N | | N | - |
| N10 | *Enterobacter* sp. | + | | N | N | | N | - |
| N11 | *Pseudomonas* sp. | + | | N | N | | N | - |
| N12 | *Pantoea* sp. | + | | N | N | | N | - |
| N13 | *Erwinia* sp. | + | | N | N | | N | - |
| P1 | *Klebsilla Pneumoniae* | N | | + | + | | N | - |
| P2 | *Klebsiella* sp. | N | | + | - | | N | - |
| P3 | *Enterobacter asburiae* | N | | + | + | | N | - |
| P4 | *Raoultella* sp*.* | N | | + | - | | N | - |
| P5 | *Klebsiella variicola* | N | | + | + | | N | - |
| P6 | *Agrobacterium* sp. | N | | + | - | | N | - |
| P7 | *Rhizobium* sp. | N | | + | - | | N | - |
| P8 | *Enterobacter* sp. | N | | - | + | | N | - |
| P9 | *Klebsiella* sp. | N | | + | + | | N | - |
| P10 | *Enterobacter* sp. | N | | - | + | | N | - |
| P11 | *Comamonas* sp. | N | | - | + | | N | - |
| K1 | *Klebsiella variicola* | N | | + | - | | + | - |
| K2 | *Enterobacter* sp. | N | | + | - | | + | - |
| K3 | *Raoultella* sp*.* | N | | + | - | | + | - |
| K4 | *Klebsiella variicola* | N | | + | - | | + | - |
| K5 | *Klebsiella variicola* | N | | + | - | | + | - |
| K6 | *Klebsilla Pneumoniae* | N | | + | - | | + | - |
| K7 | *Klebsiella* sp. | N | | + | - | | + | - |
| K8 | *Raoultella* sp*.* | N | | + | - | | + | - |
| K9 | *Raoultella* sp*.* | N | | + | - | | + | - |
| K10 | *Pseudomonas* sp. | N | | + | - | | + | - |
| K11 | *Advenella* sp. | N | | + | - | | + | - |
| K12 | *Agrobacterium* sp. | N | | + | - | | + | - |
| K13 | *Rhizobium* sp. | N | | + | - | | + | - |
| K14 | *Bacillus* sp. | N | | + | - | | + | + |
| K15 | *Microbacterium* sp. | N | | + | - | | + | - |

Note: + Positive; - Negative; N Not determination

| Table S2 Effect of bacterial combination on plant height, fresh weight and dry weight | | | | |
| --- | --- | --- | --- | --- |
|  | Combination | Plant height (Cm) | Fresh weight (g) | Dry weight (g) |
| C0 | Control | 26.14±0.48 | 2.27±0.46 | 1.00±0.25 |
| C1 | K14+176+P9+N8+K10 | 30.34±2.10 | 4.64±0.45 | 1.95±0.13 |
| C2 | K14+176+P9+N8+K12 | 24.72±1.43 | 2.71±0.51 | .88±0.18 |
| C3 | K14+176+P9+N8+K13 | 24.44±0.91 | 3.62±0.46 | .96±0.10 |
| C4 | K14+176+P9+N8+P1 | 27.57±0.83 | 2.21±0.30 | 1.07±0.13 |
| C5 | K14+176+P9+N8+P3 | 24.72±1.41 | 2.32±0.48 | 0.95±0.16 |
| C6 | K14+176+P9+N8+P5 | 34.86±1.26 | 4.48±0.42 | 1.43±0.13 |
| C7 | K14+176+P9+N9+K10 | 36.59±2.31 | 4.37±0.35 | 1.75±0.04 |
| C8 | K14+176+P9+N9+K12 | 22.96±2.87 | 2.49±0.29 | .70±0.11 |
| C9 | K14+176+P9+N9+K13 | 35.61±1.80 | 3.73±0.75 | 1.40±0.23 |
| C10 | K14+176+P9+N9+P1 | 27.91±1.18 | 2.96±0.28 | .95±0.17 |
| C11 | K14+176+P9+N9+P3 | 28.39±2.83 | 3.58±0.49 | 1.04±0.10 |
| C12 | K14+176+P9+N9+P5 | 33.33±2.48 | 4.24±0.42 | 1.37±0.06 |
| C13 | K14+176+P9+N10+K10 | 26.87±0.76 | 3.43±0.91 | 1.00±0.10 |
| C14 | K14+176+P9+N10+K12 | 27.30±1.40 | 3.48±0.71 | 1.07±0.09 |
| C15 | K14+176+P9+N10+K13 | 31.22±2.35 | 4.22±0.49 | 1.30±0.05 |
| C16 | K14+176+P9+N10+P1 | 30.45±2.72 | 4.33±0.51 | 1.34±0.10 |
| C17 | K14+176+P9+N10+P3 | 26.12±1.07 | 3.48±0.73 | .95±0.13 |
| C18 | K14+176+P9+N10+P5 | 26.41±1.97 | 3.43±0.44 | 1.10±0.1 |
| C19 | K14+176+P11+N8+K10 | 28.18±0.77 | 3.20±0.79 | .86±0.10 |
| C20 | K14+176+P11+N8+K12 | 23.70±1.21 | 2.86±0.73 | 1.03±0.39 |
| C21 | K14+176+P11+N8+K13 | 25.32±1.37 | 2.82±0.57 | 1.05±0.06 |
| C22 | K14+176+P11+N8+P1 | 24.56±1.12 | 3.26±0.52 | 1.08±0.08 |
| C23 | K14+176+P11+N8+P3 | 26.10±1.71 | 2.92±0.30 | 1.07±0.11 |
| C24 | K14+176+P11+N8+P5 | 30.73±1.38 | 3.75±0.78 | 1.28±0.11 |
| C25 | K14+176+P11+N9+K10 | 27.58±1.21 | 3.58±0.66 | 1.05±0.13 |
| C26 | K14+176+P11+N9+K12 | 25.18±1.95 | 3.21±0.67 | 1.03±0.19 |
| C27 | K14+176+P11+N9+K13 | 25.56±2.23 | 2.88±0.69 | 1.05±0.14 |
| C28 | K14+176+P11+N9+P1 | 24.47±1.81 | 2.58±0.20 | .98±0.13 |
| C29 | K14+176+P11+N9+P3 | 25.28±1.28 | 3.25±0.71 | 1.04±0.21 |
| C30 | K14+176+P11+N9+P5 | 24.53±1.06 | 2.97±0.22 | 1.11±0.14 |
| C31 | K14+176+P11+N10+K10 | 28.47±0.36 | 3.88±0.13 | .95±0.10 |
| C32 | K14+176+P11+N10+K12 | 27.16±0.75 | 2.79±0.24 | 1.08±0.12 |
| C33 | K14+176+P11+N10+K13 | 34.79±2.05 | 3.63±0.23 | 1.08±0.14 |
| C34 | K14+176+P11+N10+P1 | 31.50±1.97 | 3.42±0.69 | 1.13±0.15 |
| C35 | K14+176+P11+N10+P3 | 28.08±0.64 | 3.67±0.57 | 1.02±0.09 |
| C36 | K14+176+P11+N10+P5 | 21.81±1.46 | 2.99±0.40 | 1.04±0.13 |
